# SpirPro: A *Spirulina* proteome database and web-based tools for the analysis of protein-protein interactions at the metabolic level in *Spirulina (Arthrospira) platensis* C1

**DOI:** 10.1186/s12859-015-0676-z

**Published:** 2015-07-29

**Authors:** Jittisak Senachak, Supapon Cheevadhanarak, Apiradee Hongsthong

**Affiliations:** Biochemical Engineering and Pilot Plant Research and Development (BEC) Unit, National Center for Genetic Engineering and Biotechnology, National Science and Technology Development Agency at King Mongkut’s University of Technology Thonburi, 49 Soi Thian Thalae 25, Bang Khun Thian Chai Thalae Rd., Tha Kham, Bang Khun Thian, Bangkok 10150 Thailand; School of Bioresources and Technology, King Mongkut’s University of Technology Thonburi, 49 Soi Thian Thalae 25, Bang Khun Thian Chai Thalae Rd., Tha Kham, Bang Khun Thian, Bangkok 10150 Thailand; Pilot Plant Development and Training Institute (PDTI), King Mongkut’s University of Technology Thonburi, 49 Soi Thian Thalae 25, Bang Khun Thian Chai Thalae Rd., Bang Khun Thian, Bangkok 10150 Thailand

**Keywords:** Proteome, Database, Protein-protein interaction, *Spirulina* (*Arthrospira*), Cyanobacteria

## Abstract

**Background:**

*Spirulina (Arthrospira) platensis* is the only cyanobacterium that in addition to being studied at the molecular level and subjected to gene manipulation, can also be mass cultivated in outdoor ponds for commercial use as a food supplement. Thus, encountering environmental changes, including temperature stresses, is common during the mass production of *Spirulina*. The use of cyanobacteria as an experimental platform, especially for photosynthetic gene manipulation in plants and bacteria, is becoming increasingly important. Understanding the mechanisms and protein-protein interaction networks that underlie low- and high-temperature responses is relevant to *Spirulina* mass production. To accomplish this goal, high-throughput techniques such as OMICs analyses are used. Thus, large datasets must be collected, managed and subjected to information extraction. Therefore, databases including *(i)* proteomic analysis and protein-protein interaction (PPI) data and *(ii)* domain/motif visualization tools are required for potential use in temperature response models for plant chloroplasts and photosynthetic bacteria.

**Descriptions:**

A web-based repository was developed including an embedded database, SpirPro, and tools for network visualization. Proteome data were analyzed integrated with protein-protein interactions and/or metabolic pathways from KEGG. The repository provides various information, ranging from raw data (2D-gel images) to associated results, such as data from interaction and/or pathway analyses. This integration allows *in silico* analyses of protein-protein interactions affected at the metabolic level and, particularly, analyses of interactions between and within the affected metabolic pathways under temperature stresses for comparative proteomic analysis. The developed tool, which is coded in HTML with CSS/JavaScript and depicted in Scalable Vector Graphics (SVG), is designed for interactive analysis and exploration of the constructed network. SpirPro is publicly available on the web at http://spirpro.sbi.kmutt.ac.th.

**Conclusions:**

SpirPro is an analysis platform containing an integrated proteome and PPI database that provides the most comprehensive data on this cyanobacterium at the systematic level. As an integrated database, SpirPro can be applied in various analyses, such as temperature stress response networking analysis in cyanobacterial models and interacting domain-domain analysis between proteins of interest.

## Background

Cyanobacteria have been experimentally used as a model for plants and bacteria, especially for the study of photosynthesis [1], due to their similarity in biochemistry to the microorganism thought to be the precursor of chloroplasts. Moreover, cyanobacteria are easily genetically manipulated and rapidly grown in liquid culture, which makes it easy to scale up their production in photobioreactors. Therefore, many cyanobacteria, such as *Synechocystis sp.*, *Synechococcus sp.,* and *Spirulina (Arthrospira) platensis*, have been studied at the genomic and proteomic levels.

In addition to its wide recognition due to its use in food supplements, *Spirulina* is the only cyanobacterium that can be mass cultivated in outdoor ponds, where fluctuating environmental temperatures can cause unwanted effects on biomass yields and cell contents. Thus, investigation of the high- and low-temperature response mechanisms of *Spirulina* was performed through proteomic analyses.

Large datasets for *Spirulina (Arthrospira)* obtained using high-throughput techniques at the genomic and proteomic levels have been previously collected [2–8]. Thus, databases and bioinformatics tools have been constructed to explore and to conduct in-depth studies of the raw data obtained from these high-throughput techniques. Once the *Spirulina* genome sequence became available [4], proteomic analyses of *Spirulina* under optimal and temperature stress conditions were conducted by our research group [6–8].

Furthermore, a recent study by our group focused on comparative proteomic analyses of low- and high-temperature stresses and potential protein-protein interaction networks, constructed using a bioinformatics approach, in response to both types of stress conditions [8]. The data revealed linkages between temperature stress and other mechanisms within cells (e.g., nitrogen and ammonia assimilation and signaling pathway cross-talk). Among these potential protein networks, chaperones were observed within the central PPI network hubs.

Extreme environmental temperature change is currently one of the problems being caused by global warming and is leading to more negative effects on the mass cultivation of cells in outdoor systems. Thus, understanding the temperature stress response at the systematic level is relevant. In this work, SpirPro, an integrated database for *Spirulina,* was developed. This database provides possible mechanisms, in term of PPI networks and proteome-wide expression levels, underlying the temperature stress response of this cyanobacterium for use as model mechanisms for other photosynthetic organisms, such as plants, algae and other cyanobacteria. Moreover, proteome-wide domain identification is available in the database, which might be useful for further studies on protein-protein interaction domain analyses.

## Construction and content

SpirPro was constructed as a useful resource for examining proteomic effects on protein-protein interaction networks and metabolic pathway studies. At the front-end, web-based tools were developed, with a user-friendly web interface for information queries and retrieval from the back-end database. In the present work, we collected all available multi-level data on *Spirulina (Arthrospira) platensis* strain C1, which currently consist of the genome, quantitative proteome data and phosphoproteome and interactome data. The *Spirulina* genome and proteome were obtained from our previous work [4, 6–8], whereas other cyanobacterial genomes were retrieved from NCBI [9].

**Fig. 1:**
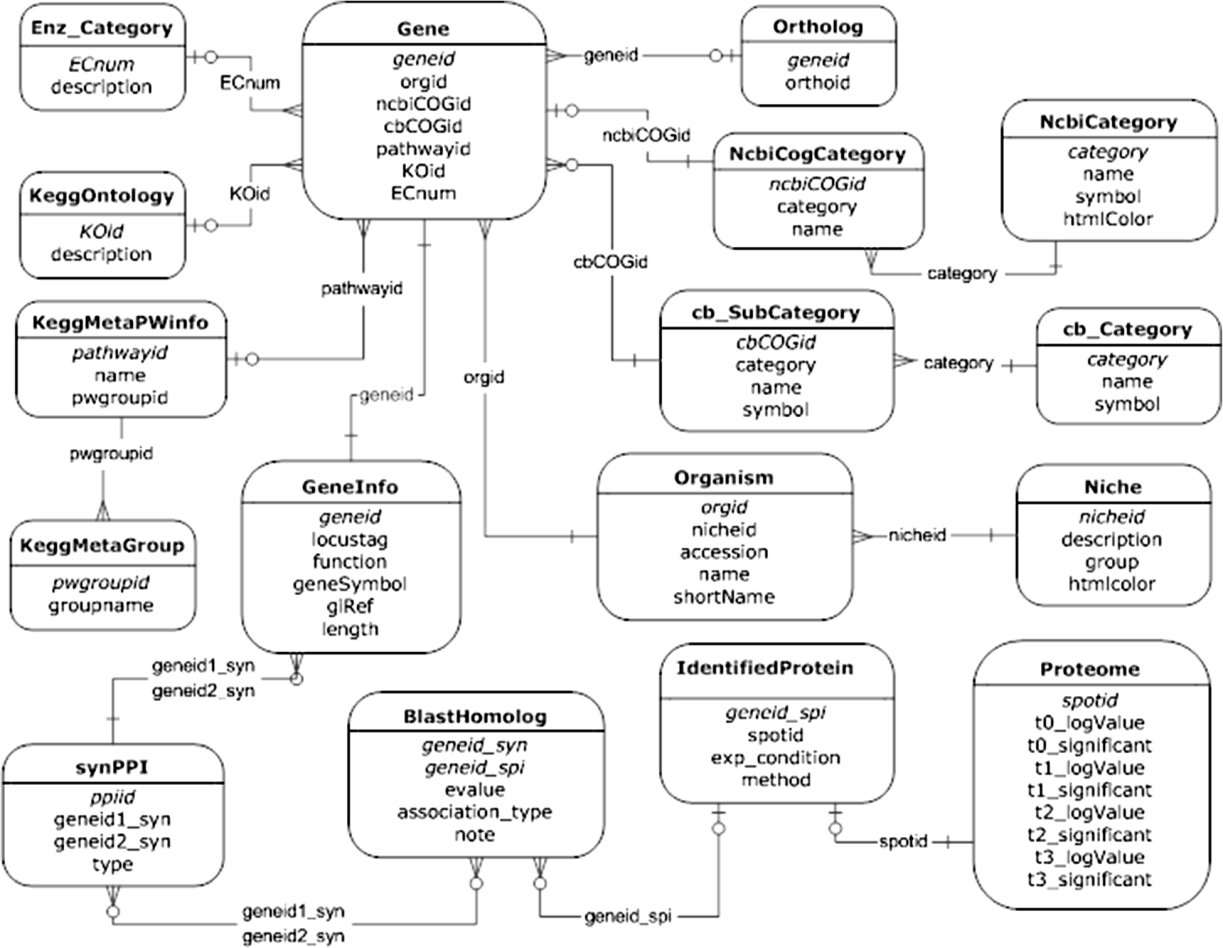
Database schematic diagram for the SpirPro website (17 tables)

Based on our quantitative proteomic data, interactome data were computationally generated through inference from orthologous proteins involved in PPIs in another cyanobacterium, *Synechocystis* sp. PCC 6803. Moreover, pathway information associated with the *Spirulina* prototype PPI network was incorporated into the database obtained from KEGG pathways. All of the data in SpirPro were organized into 17 relational tables and maintained with MySQL, as depicted in Fig. 1.

Moreover, SpirPro provides various visualization tools in the menu that were designed for interactive analysis and network exploration of the integrated data across multi-level data, including genome, proteome, interactome and metabolic pathways. The details regarding the contents of the integrated database and how we constructed it are provided in this section, organized into 4 subsections (A-D), as follows.

(A) Genome-scale data, protein orthologs and domain visualization

The cyanobacterial model organism *Synechocystis* sp. PCC6803 was chosen as reference, or template genome for our analyses due to the complete genome-scale information available for this species. The complete genomes of *Synechocystis* and *Spirulina* were retrieved from the NCBI website and our previous work, respectively. Groups of protein orthologs were identified through reciprocal Blast analysis with the default parameters and E-values under the threshold of 1e-10. Proteins in the same orthologous group were identified and assigned an identifier, which we referred to as the ortholog-id, using the OrthoMCL algorithm and tool [10]. Furthermore, to reconfirm that all groups of protein orthologs shared the same functions, protein domain analysis was carried out with the PfamScan program, against the PFam database v.24 [11]. For further analyses and data integration, a database of all of the analyzed data was constructed.

Under the hypothesis that proteins with similar sequences (orthologous) and domains may share the same function, we reconfirmed all of the protein orthologs through protein domain analysis using our in-house web-based tool CyanoCOG. Taking ortholog-ids, ORF names, gene symbols, function descriptions, or even words in COG function categories as input keywords, the tool performs searches against the constructed database and returns a list of all keyword-matched genes or information on proteins. It also provides a feature in which the protein domains of designated proteins can be visualized on the output page. This feature facilitates comparisons of protein domains and analyses of protein orthologous groups, which allows functions to be assigned to groups of protein orthologs.

(B) Interactions and network construction

PPI networks for *Spirulina* were constructed using *Synechocystis* interaction data from Cyanobase [12, 13], which were obtained experimentally using the yeast two-hybrid system [14]. The interactions retrieved from Cyanobase were employed as the template for the *Spirulina* PPI network. In the present study, orthologous proteins and their *Synechocystis*-inferable interactions were used for the construction of a *Spirulina* prototype PPI network, which was represented as graph nodes and edges, respectively

(C) Proteome-scale data and integration

The *Spirulina* prototype PPI network was enriched with our previous proteomic data obtained under temperature-stress conditions. Under conditions of a growth temperature up-shift (35 °C to 40 °C) or down-shift (35 °C to 22 °C), two proteomic techniques (two-dimensional differential gel electrophoresis/mass spectrometry (2D-DIGE) [6, 7] and liquid chromatography/tandem mass spectrometry (LC-MS/MS) [8]) were applied to identify and quantitate differentially expressed proteins. The data from 2D-DIGE were factorized according to two different pH ranges (3–10 and 4–7) and three sub-cellular fractions (the plasma membrane (PM), thylakoid membrane (TM) and cytosol (Cyt)).

(D) Metabolism-level data and integration

To obtain biological meaning in metabolic scale, *Spirulina* proteins in proteome-enriched PPI network were integrated and highlighted in all possible KEGG metabolic pathways by using the KEGG Mapper tool [15, 16] and given Synechocystis’s locus tags, which are orthologous to Spirulina proteins, as the input. The proteome-mapped pathway information was exported in HTML format with an embedded static image of the pathways. Moreover, the static web pages were simplified and re-organized to fit our web interface, and these interactive analytical tools therefore became more user-friendly.

## Utility and Discussion

At present, SpirPro provides 1659 predicted interactions across 417 *Spirulina* proteins based on 2199 experimental interactions across 1167 *Synechocystis* proteins screened using the yeast two-hybrid system. Moreover, 4804 proteins (covering 79 % of the *Spirulina* genome) identified under two different temperature-stress conditions and optimal temperature conditions are shown in 12 images obtained through 2D-gel analyses. A set of 144 differentially expressed proteins were mapped onto the PPI template and onto 97 KEGG pathways. Thus far, we have developed six tools to facilitate interactive analyses of proteomic data.

1. **2D-gel Images**; The tool provides proteomic results, in the form of intensity images of protein spots (Fig. 2 – upper panel), and a list of the differentially expressed proteins identified from spots according to peptide mass fingerprints (Fig. 2 – lower panel), based on our experiments using two-dimensional difference gel electrophoresis (2D-DIGE). Each spot represents a protein visualized under a designated experimental condition.

**Fig. 2:**
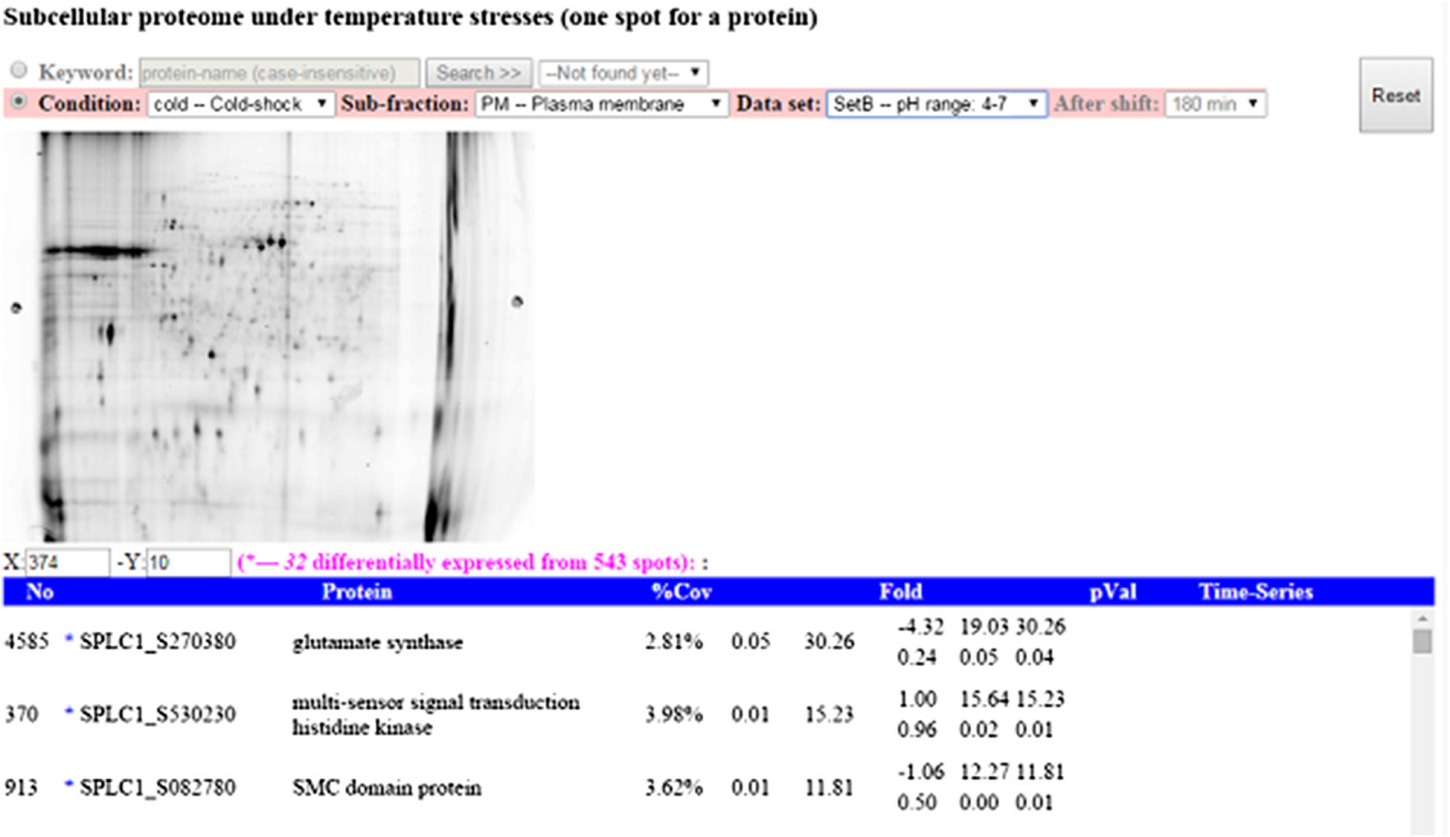
Example of using 2D-gel image tool to depict proteome results from selected condition. A list of identified proteins from protein spots (black spot in the image) is shown in the table, sorted by absolute value of fold-change. The asterisk marks before the ORF names denote such proteins that are differentially expressed
2. **Snapshot Interactions,** The tool displays protein-protein interactions around a particular protein as a network graph as well as a list of proteins and detailed information on these proteins, as shown in Fig. 3. It may be used for analysis via network exploration to suggest a signaling pathway from an expressed protein after perturbation to others.

**Fig. 3:**
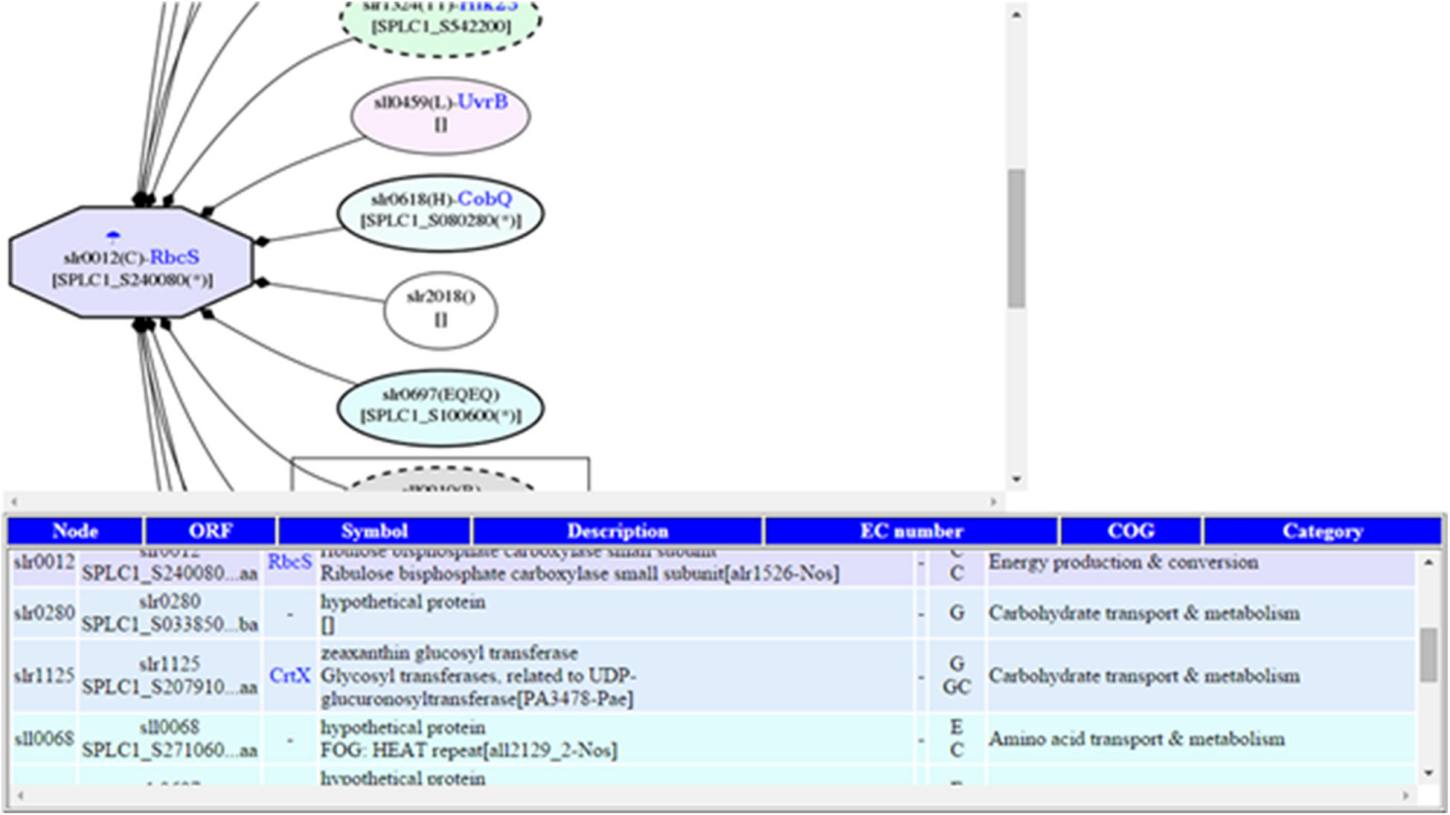
Example of using Snapshot-Interaction tool to visualize *(i)* the PPI network around RbcS protein [SPLC1_S240080] of *S. platensis* C1, which is orthologous to the slr0012 protein of *Synechocystis sp.* PCC 6803, and *(ii)* differentially expressed proteins after temperature down-shift in cytosol fraction. Note: A node marked by an octagon represents differential expression. In the node label, line 1 describes protein expression conditions and fractions (with colored marks); line 2 shows the corresponding *Synechocystis* ORF and gene name (blue text); and line 3 shows protein ortholog in *Spirulina*. The mark denotes expressed conditions: temperature up-shift (sun) and down-shift (umbrella/snow-man). The colors of the marks, blue, green and orange, represent the cytosol fraction, thylakoid membrane fraction, and plasma membrane fraction, respectively
3. **Inter-pathways**; The tool shows protein-protein interactions across two specified pathways. As depicted in Fig. 4, the interactions and details are listed in the table located in the middle frame between the images of the two pathways whose proteins are listed in the interactions. The analysis of inter-pathway interactions may reveal possible regulatory pathways at the metabolic level, i.e., from the differentially expressed proteins to other proteins under certain temperature stress conditions.

**Fig. 4:**
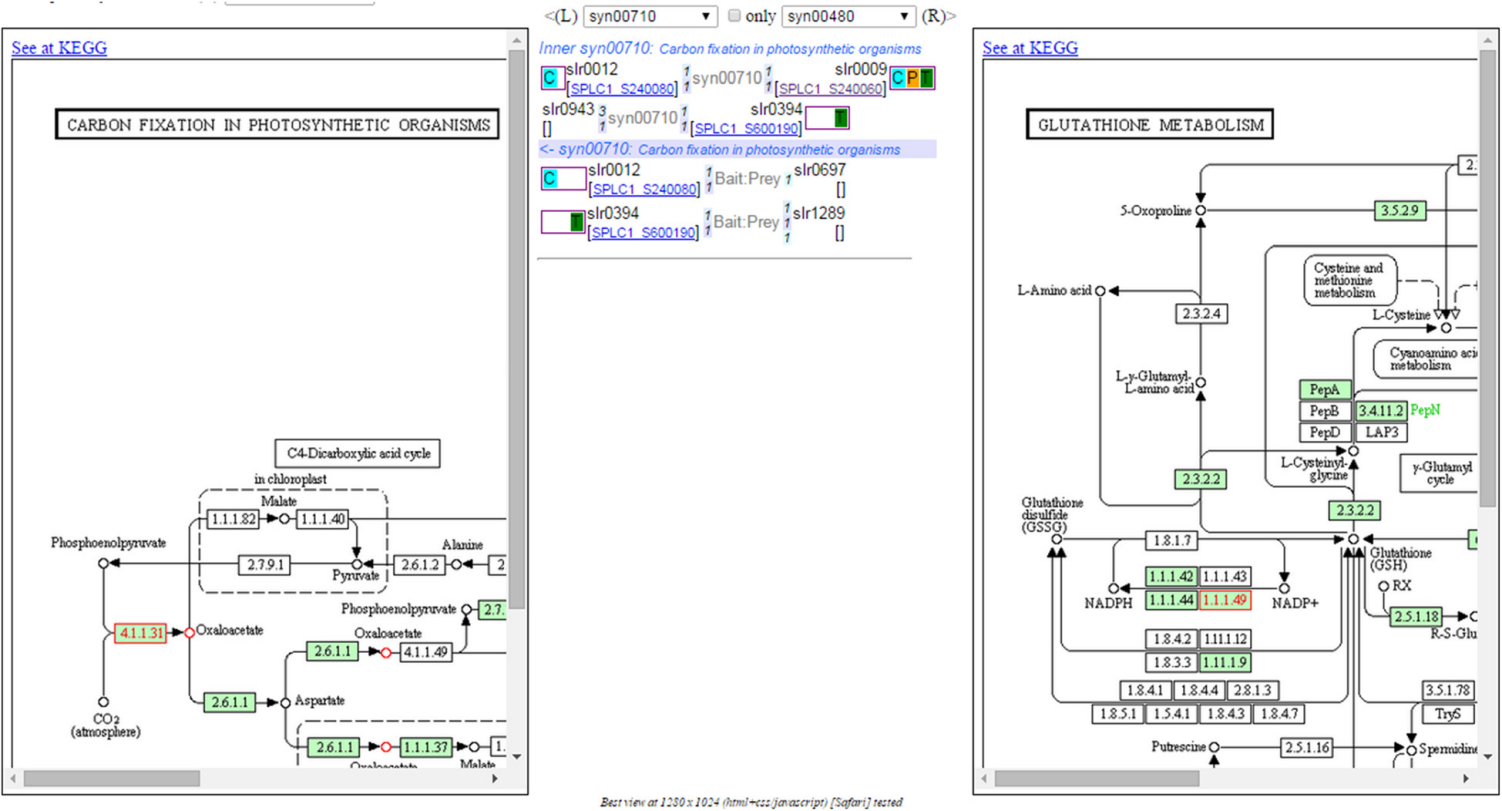
Example of using “Inter-pathways” tool to query interactions between two pathways (left─syn00710: Carbon fixation; right─syn00480: glutathione metabolism). The interaction details between the two pathways show that RbcS protein [SPLC1_S240080] can interact with RbcL protein [SPLC1_S240060] and with slr0697-ortholog protein in glutathione metabolism. (The pathway image files were generated from KEGG’s Color Pathway tool by inputting our lists of differentially expressed proteins in each pathway)
4. **Effect on Metabolisms**; The tool highlights expressed proteins in a specified pathway, as shown in Fig. 5. It allows users to perform comparative analyses among proteins expressed under different stress conditions; e.g., housekeeping genes may be expressed in any conditions, whereas heat shock proteins are expressed under high-temperature conditions.

**Fig. 5:**
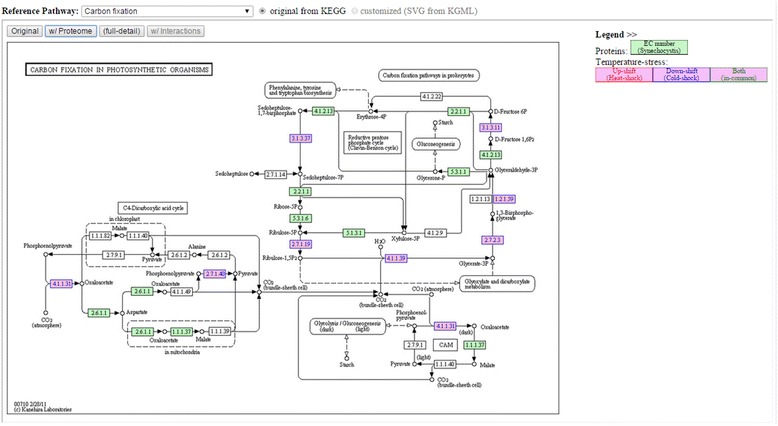
Example of using “Effect on Metabolism” tool to see the overall proteome result mapped on carbon fixation metabolism. Note: Numbers in blue, red (not shown in this figure) and green (not shown in this figure) represent proteins found under temperature down-shift, up-shift and both up/down-shift conditions, respectively. (The pathway image files were generated from KEGG’s Color Pathway tool by inputting our lists of differentially expressed proteins)
5. **YTH Experiments**; Protein-protein interactions of interest from *Synechocystis* were selected. The yeast two-hybrid technique was employed for the designated *Spirulina* proteins to verify the bioinformatics data obtained from the *Synechocystis* database. The results are depicted as a network graph in Fig. 6.

**Fig. 6:**
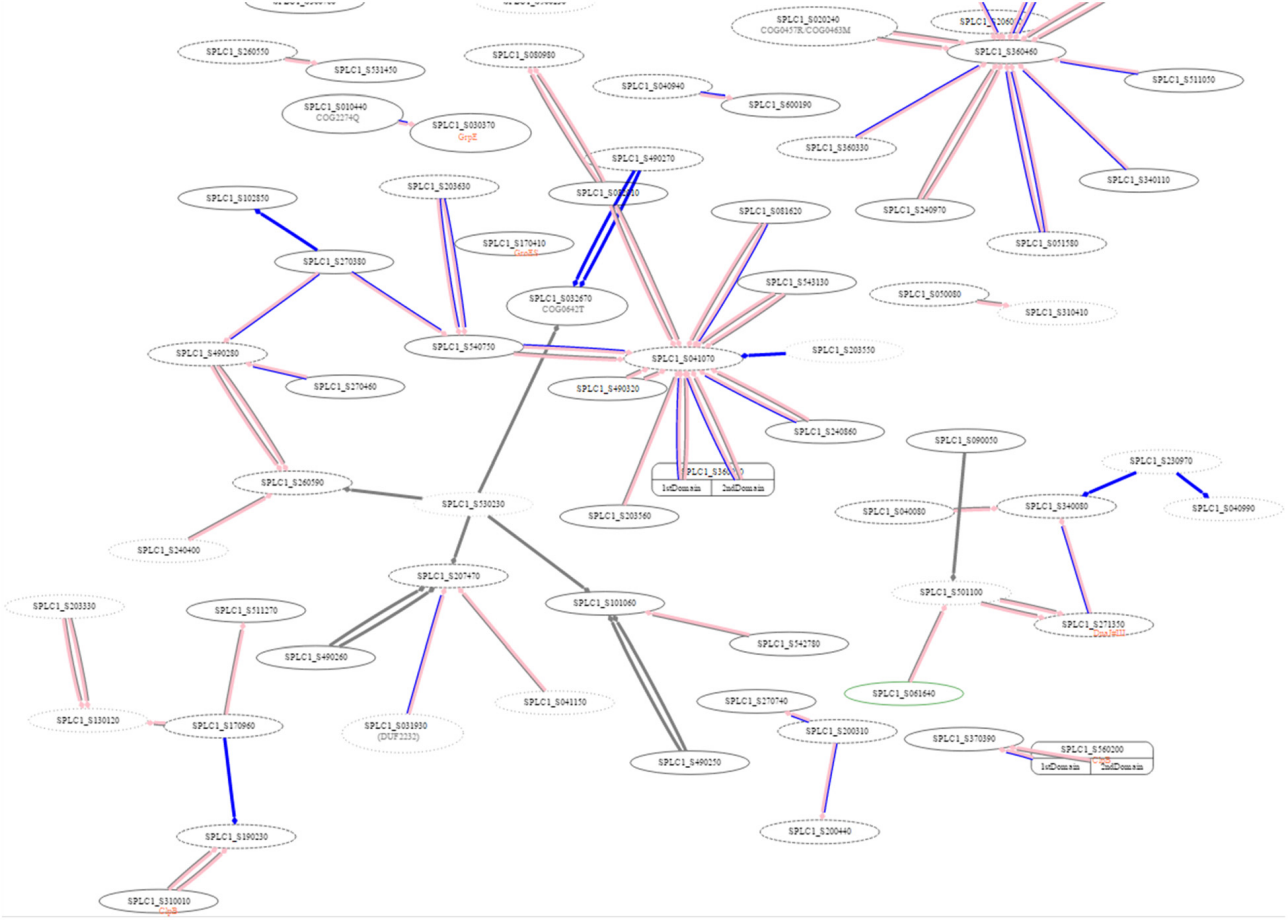
Snapshot of using “YTH experiment” tools to visualize our experimentally verified interaction in *S. platensis* C1 using the yeast two-hybrid system. All nodes are *Spirulina* proteins. The pink, blue and grey edges are PPI inferred from *Synechocystis*, positive bait-prey pair of *S. platensis*, and negative bait-prey pair of *S. platensis*, respectively
6. **CyanoCOG**; In Fig. 7, when a given protein of interest is used as a keyword in the query box, the tool can perform matching using either protein names or properties. The output page provides a list of all keyword-matched genes with detailed information, including their domain structures. Moreover, multi-sequence alignment of protein orthologs among cyanobacteria can be viewed by clicking on < msa > under ortholog-id.

**Fig. 7:**
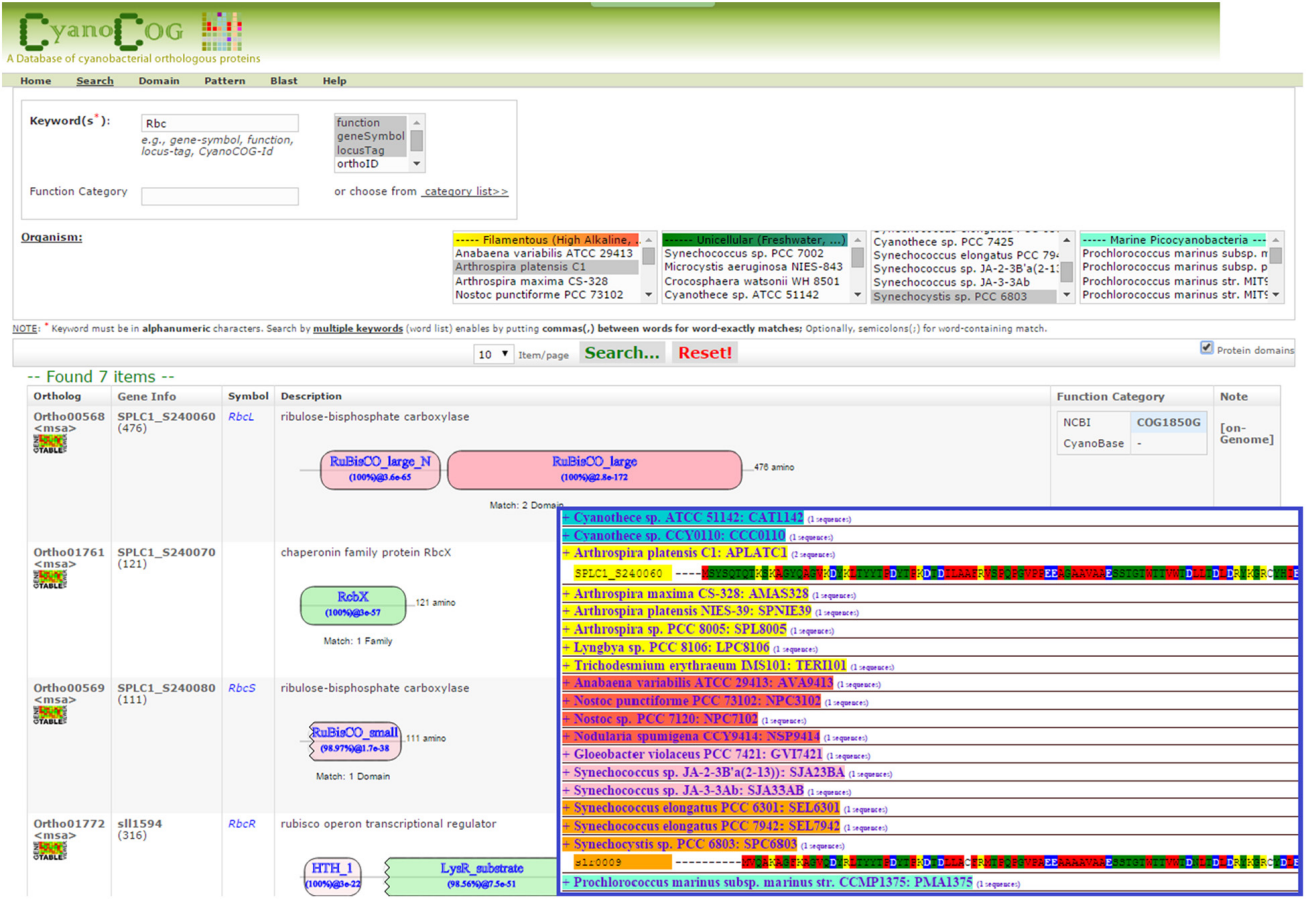
Example of using “CyanoCOG” to query Rbc proteins in *S. platensis* C1 and *Synechocystis*. Detail of domain in all matched proteins will be displayed by ticking at “protein domain” on the right under search button. Moreover, multiple sequence alignments of all proteins in a specified ortholog will be visualized by clicking “<msa>” label under ortholog-id

Post-genomics level analyses are becoming an increasingly important approach for exploring the extreme complexity of cellular responses and regulation. Bioinformatics tools are essential for these types of complicated analyses. The tools and databases described in the present report can function as a platform not only for collecting and managing high-throughput datasets but also for extracting important information from the complex datasets. For example, in terms of metabolism, analyses performed using the “inter-pathway” and “effect on metabolisms” tools illustrated the effect of low- and high-temperature stresses on several pathways, including photosynthesis, nitrogen metabolism, protein and amino acid biosynthesis, fatty acid biosynthesis and carbohydrate metabolism.

## Conclusions

A platform for integrative genome and proteomic data analysis was developed using the available genome- and proteome-scale data for *S. platensis* strain C1 (wild type) as a model in comparison with data obtained from Cyanobase. We developed a data repository integrated with an analysis support tool and provided following data: 1) raw image results and proteins identified through proteomic analyses conducted under temperature stress conditions and optimal growth conditions; 2) protein-protein interactions around proteins in interest; 3) data from *in silico* analyses of interactions between and within affected metabolic pathways; 4) interactions and overall metabolism; and 5) certain interactions that have been experimentally verified using the yeast two-hybrid technique. A visualization tool with embedded data facilitates biological demonstration of the stress effects on the cells via networking interactions, which will be useful for further in-depth analyses of the mechanisms and regulation of cellular stress responses.

## Availability and requirements

SpirPro was developed as a web database tool running on Apache HTTP Server with MySQL database server (version: 5.0). All data entries are stored and maintained as a MySQL database. Each tool in the menu was coded in HTML using the jQuery JavaScript Library (version: 1.5.1) and Cascading Style Sheets (CSS) for web design for interactive use. The Scalable Vector Graphics (SVG) format was preferred to visualize the networks and allow dynamic functions of picture panning and zooming. The web server and database are available at http://spirpro.sbi.kmutt.ac.th.

## Abbreviations

ORF: Open reading frame
PPI: Protein-protein interaction
KEGG: Kyoto encyclopedia of genes and genomes
KGML: KEGG markup language
2-DIGE: Two-dimensional difference gel electrophoresis
YTH: Yeast two-hybrid system screen
HTML: Hypertext markup language
CSS: Cascading style sheets
SVG: Scalable vector graphics
